# Perceived Stress Is Linked to Depression but Not to Clinical Outcomes in Maintenance Hemodialysis Patients

**DOI:** 10.3390/jcm14030883

**Published:** 2025-01-29

**Authors:** Maurizio Bossola, Laura Angioletti, Marta Di Giovanni, Ilaria Mariani, Enrico Di Stasio, Michela Balconi

**Affiliations:** 1 Servizio Emodialisi, Università Cattolica del Sacro Cuore, 20123 Rome, Italy; mauriziobossola@gmail.com; 2 Faculty of Medicine, Fondazione Policlinico Universitario A. Gemelli IRCCS, 00168 Rome, Italyilaria.mariani04@icatt.it (I.M.);; 3 International Research Center for Cognitive Applied Neuroscience (IrcCAN), Università Cattolica del Sacro Cuore, 20123 Milan, Italy; 4 Divisione di Nefrologia, Università Cattolica del Sacro Cuore, 00168 Rome, Italy; 5 Divisione di Chimica, Biochimica, E Biochimica Molecolare, Università Cattolica del Sacro Cuore, 00168 Rome, Italy

**Keywords:** perceived stress levels, depression, ESRD patients, hemodialysis, gender

## Abstract

**Background/Objectives**: Over the course of end-stage renal disease, patients undergoing hemodialysis (HD) often face significant psychological distress. Nonetheless, little is known about perceived stress levels and related factors in HD patients. This is a cross-sectional study that explores the prevalence of perceived stress levels and the associated variables in HD patients. **Methods**: Participants included 223 HD patients recruited in June 2024 in Italy. Perceived stress and depression levels were measured with the Perceived Stress Scale (PSS) and Beck Depression Inventory (BDI-II). We also collected clinical and laboratory variables to evaluate their association with PSS. **Results**: PSS score was moderate in 70.8% and high in 11.2% of the patients. The BDI score was significantly higher in patients with moderate or high perceived stress than in those with low perceived stress. The correlation between PSS and BDI scores was statistically highly significant, and in multivariate regression analysis, PSS score was independently associated with BDI, but not with age, sex, and serum creatinine. Patients with moderate or high perceived stress more frequently had a BDI ≥ 17. In women, with respect to men, the frequency of high and moderate perceived stress was higher. PSS does not correlate with some clinical characteristics such as functional disability (ADL and IADL scores), and the number of comorbidities (Charlson comorbidity Index). Also, we found that there was no correlation between PSS and post-dialysis fatigue prevalence/characteristics, nor between PSS and time of recovery after dialysis. **Conclusions**: These findings emphasize the critical need for targeted interventions addressing stress management in HD patients, especially with gender-specific approaches.

## 1. Introduction

Worldwide, approximately three million people receive renal replacement therapy (RRT) because of end-stage renal disease (ESRD) [1]. With an estimated prevalence of 465 and 1437 per million people in Europe and the U.S., respectively, hemodialysis (HD) is by far the most common form of RRT [1]. Throughout the progression of end-stage renal disease (ESRD), patients undergoing HD often face significant psychological distress [2,3,4]. Unfortunately, little is known about perceived stress levels and related factors in ESRD patients receiving maintenance HD.

Perceived stress refers to the thoughts and feelings an individual has regarding the level of stress they are experiencing at a specific moment or over a certain time period. It encompasses perceptions of the uncontrollability and unpredictability of life, the frequency of dealing with difficult challenges, the amount of change one is undergoing, and their confidence in managing these problems or difficulties. Rather than assessing the types or frequency of stressful events, it concerns how a person feels about the overall stressfulness of their life and their capacity to cope with it [5].

The gold standard instrument for measuring stress perception is the Perceived Stress Scale (PSS) [6] that assesses the degree to which a respondent finds circumstances in their life to be unpredictable, uncontrollable, and/or overwhelming and, in contrast to measures that assess environmental stress exposures, captures a respondent’s subjective appraisal of whether life circumstances and experienced events exceed their adaptive capacity [7].

A growing body of evidence suggests that perceived stress is a common experience among patients affected by chronic diseases such as cancer, multiple sclerosis, Parkinson’s disease, diabetes, and HIV infection/AIDS [8,9,10,11,12,13] and may lead to persistent and more severe anxiety, depression, and pain [14,15]. Moreover, the prevalence of depression among patients on HD is over four times higher than in the general population, driven by a complex interplay of physiological, behavioral, and gender factors [16]. Therefore, there is a need to address perceived stress and its relation to depression among HD patients.

Given these premises, the current research sought to define the prevalence and levels of perceived stress in a sample of patients on maintenance HD and to investigate the demographic (age, gender, weight, height, and body mass index, BMI), clinical (primary cause of renal disease, dialytic age, Charlson comorbidity score index, functional disability, depression levels measured with the Beck Depression Inventory [17], interdialytic weight gain, post dialysis fatigue parameters, recovery time after the HD session), and laboratory (Serum albumin, Serum Creatinine, Hemoglobin, and Hematocrit) associated variables.

## 2. Materials and Methods

### 2.1. Patients

All currently chronic patients referred to the HD unit in June 2024 were considered eligible for this study. According to the calculation formula for the cross-sectional survey, when α =  0.05, the corresponding Z is 1.96. In absence of prior meta-analysis and studies on Italian samples, we based our estimation on Tao and colleagues’ prior study, which revealed a prevalence of 13.5% of excessive stress in hemodialysis patients [2]. So, *p* was set to 0.135 in this study, and the allowable error E was set to 0.05. The sample size required for this method was 179 patients. Accounting for a 10% non-response rate, the final adjusted sample size was 199.

Exclusion criteria were as follows: dialysis duration < 1 year due to physical and psychological instability, inability to answer the questionnaires because of hearing or reading problems, diagnosis of dementia based on DSM-IV criteria, presence of acute infectious disease(s), active cancer, or active cancer treatment. Informed consent was obtained by each patient included in the study. A total sample of 223 patients was included in the study.

The following demographic, clinical, and laboratory data were recorded for each patient at the moment of inclusion in the study: age, gender, primary cause of renal disease, dialytic age, weight, height, BMI, comorbidity through the Charlson comorbidity score index [18], activity of daily living (ADL) [19], instrumental activity of daily living (IADL) [20], serum albumin, creatinine, hemoglobin, hematocrit, weigh gain, Na concentration in the dialysate, temperature of dialysate, and Kt/V.

Blood samples were obtained after overnight fasting from patients through the arteriovenous fistula or the central venous catheter immediately before their scheduled HD session at the beginning of the week. The plasma was separated within 30 min, and samples were kept frozen at −70 °C if not analyzed immediately. Laboratory parameters were measured by routine methods at the Department of Laboratory Medicine, Policlinico Universitario A. Gemelli IRCCS, Rome, Italy.

### 2.2. Type of HD

Patients were receiving 4-h bicarbonate HD or online hemodiafiltration (HDF), three times a week. The dialysis treatment duration was 240 min. In HD, the blood flow ranged from 250 to 300 mL/min with a dialysis flow rate of 500 mL/min. In HDF, the blood flow ranged from 300 to 350 mL/min with a dialysis flow rate of 600 mL/min. All patients were treated with high-permeability membranes. Membranes were not reused. HDF was performed with a target convection volume of 22 L/treatment.

### 2.3. Assessment of Perceived Stress Levels

Perceived stress was measured with the Italian version of the Cohen’s Perceived Stress Scale [6,21]. The scale consists of 10 items, each rated on a five-point rating scale from “never” (0) to “very often” (4). Individual scores on the PSS can range from 0 to 40, with higher scores indicating higher perceived stress. Scores ranging from 0 to 13 would be considered low stress. Scores ranging from 14 to 26 would be considered moderate stress. Scores ranging from 27 to 40 would be considered high perceived stress.

### 2.4. Assessment of Symptoms of Depression

The presence and degree of depression was assessed by using the Italian version of the Beck Depression Inventory-II (BDI-II) [17]. The BDI-II is a 21-item, patient-rated scale that has been validated in the hemodialysis population. Scores can range from 0 to 63, with higher scores indicating more severe depression. The cut-off score to classify for depression was taken at a value of 14, following the guidelines to interpret the BDI-II [22].

### 2.5. Assessment of PDF and Recovery Time After Dialysis

The assessment of PDF was conducted according to the recommendations by Sklar and colleagues [23,24,25]. Each patient was interviewed during one regularly scheduled treatment. Patients were suffering from PDF if they spontaneously offered this complaint when asked the open-ended question: “Do you feel fatigued after dialysis?” If yes, then, each patient was invited to rate the intensity, duration, and frequency of PDF from 1 to 5. Intensity was defined as the magnitude of fatigue, duration as the length of time that fatigue lasted, and frequency as the number of times that fatigue happened. The recovery time after the HD session (TIRD) was calculated according to Lindsay and colleagues [26]. Briefly, patients were invited to answer the following single open-ended question: “How long does it take you to recover from a dialysis session?” Responses were subsequently converted into the number of minutes.

The questionnaires were distributed after written informed consent was obtained. Patients completed the questionnaires by themselves under the guidance of the investigators during the dialytic session.

### 2.6. Statistical Analyses

Statistical analysis was performed by using the Statistical Package for Social Science (SPSS), release 15.0. All data were first analyzed for normality of distribution using the Kolmogorov–Smirnov test of normality. Data are presented as means ± standard deviation (SD) or medians [95% CI for the median], as appropriate. A *p*-value of less than 0.05 was considered significant. The appropriate parametric (ANOVA) or non-parametric test (χ^2^ test and Friedman ANOVA) was used to assess significance of the differences between groups. A correlational analysis was conducted between the PSS score and continuous variables that are significant in the ANOVA. Multivariable logistic regression was used to assess the association between PSS score and demographic, clinical, and laboratory variables. Chi-square analysis (χ^2^) was applied to determine differences in the frequency of PSS between men and women.

## 3. Results

Two hundred twenty-three (223) patients were studied. Their demographic, clinical, and laboratory characteristics are shown in Table 1.

**Figure 1:**
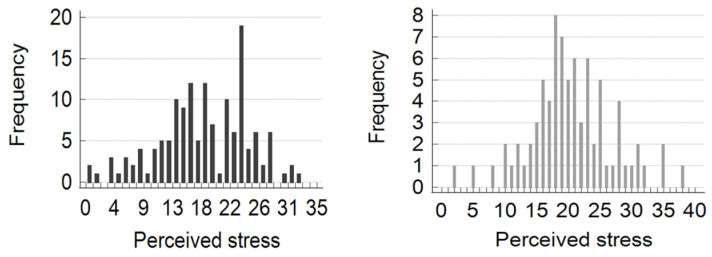
Distribution of patients according to the perceived stress scale (PSS) score. Left panel: males. Right panel: females.

Perceived stress was low in 40 (17.9%) patients, moderate in 158 (70.8%), and high in 25 (11.2%). The distribution of patients according to the PSS score and sex is shown in Figure 1. The demographic, clinical, and laboratory characteristics of the three groups divided by PSS level are shown in Table 2.

**Figure 2:**
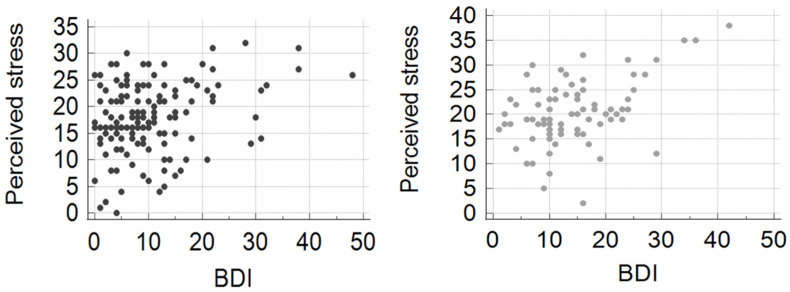
Correlation between BDI and perceived stress scale (PSS) scores. Left panel: males; correlation coefficient: 0.2946, *p* = 0.0003. Right panel: females; correlation coefficient: 0.4487, *p* < 0.0001.

The BDI score was significantly higher in patients with moderate or high perceived stress than in those with low perceived stress. The correlation between PSS and BDI scores was found to be statistically highly significant both in males and in females (Figure 2).

On the basis of the results obtained from the comparisons of the three PSS groups (results reported in Table 2), specific selected variables were included in the multivariate analysis: sex and BDI (significant results), age, and serum creatinine (results with *p*-value close to threshold of significance). In multivariate regression analysis, perceived stress was independently associated with BDI, but not with age, sex, and serum creatinine (Table 3).

As shown in Table 4, patients with moderate or high perceived stress more frequently had a BDI ≥ 17 (*p* = 0.004). However, stratifying for sex, we found that only male patients with moderate or high perceived stress more frequently had a BDI ≥ 17 (*p* = 0.005), while female patients did not (*p* = 0.098).

In women, with respect to men, the frequency of high (16.4% vs. 8.3%) and moderate (72.1% vs. 70.1%) perceived stress was higher (Chi-squared text for trend: *p* = 0.014).

## 4. Discussion

The current study demonstrated that HD patients report moderate or high perceived stress levels, measured with the PSS. The frequency of high/moderate perceived stress was found to be higher in women than in men. Moreover, the PSS score was strongly and independently associated with depression levels measured with the BDI.

As a first result, it was found that a high percentage of HD patients had moderate or high perceived stress, of 70.8% and 11.3%, respectively. Increasing evidence indicates that perceived stress is a prevalent experience among individuals with chronic illnesses [8,9,10,11,12,13]; however, relatively few studies documented the prevalence of perceived stress in HD.

Perceived stress arises when individuals perceive their environment as uncontrollable or overwhelming, which can negatively affect their well-being [27]. Patients undergoing maintenance HD experience substantial psychological stress during the course of the disease and its treatment, requiring them to adapt to such challenging circumstances [28,29]. Tao and colleagues (2023) showed that the PSS score is generally high in Chinese HD patients, with 54.8% of the patients experiencing high stress and 13.5% experiencing excessive stress [2].

Moreover, Mai and colleagues (2024) found that perceived stress is a risk factor for social isolation in HD patients of young and middle age [4]. The resource conservation theory proposes that higher levels of perceived stress lead patients to adopt self-protective coping strategies, often by limiting interactions with others. Social isolation is the most common self-protective mechanism and negative coping strategy used by vulnerable groups to cope with stress [30]. Thus, together with these findings, this study underscores the importance of addressing perceived stress in HD patients.

Secondly, the frequency of high/moderate perceived stress was higher in women than in men in the current sample of HD patients. A study conducted in Taiwan found that women undergoing HD reported higher stress levels related to physical symptoms and vascular problems compared to men [31]. In another study conducted in 414 Greek HD patients [32], higher levels of anxiety and depression were observed in female patients compared to their male counterparts: contributing factors included interpersonal stress, hormonal factors, societal roles, cultural restrictions, and social status. To the best of our knowledge, this is the first time that perceived stress and its prevalence has been highlighted in women on HD, suggesting their vulnerability and a critical need for gender-specific approaches in managing stress among HD patients, considering the unique challenges faced by women.

Thirdly, perceived stress appeared to be strongly and independently associated with depression levels measured with the BDI.

On a physiological level, oxidative stress is a key factor contributing to neurodegeneration and has been linked to the development of major depressive disorder (MDD) in the general population [33]. From a psychological perspective, individuals with MDD who exhibit greater irritability or atypical symptoms tend to report higher levels of perceived stress [34]. Both oxidative stress [35] and interpersonal stress [32] have been identified as risk factors for depression in HD patients. However, to the best of our knowledge, there is not yet a study that made a comparison between stress types and determined whether perceived stress or other types of stress have a stronger association with depression symptoms in these patients.

In HD patients, the connection between perceived stress and depression has been observed but remains insufficiently explored. One study involving 102 chronic kidney disease patients undergoing HD in Poland identified a significant positive correlation between higher perceived stress levels and increased depression, as measured by the PSS and BDI scales, respectively [36]. Other research has indicated that HD patients face elevated PSS levels due to factors like unemployment or being homemakers, compared to those who are employed or retired [3]. This evidence underscores the need to address stress perception and its relation to other psychological and psychiatric symptoms among HD patients.

Interestingly, with reference to the relation between psychological findings and clinical or functional markers relevant to patients undergoing HD, we have demonstrated that PSS does not correlate with some clinical characteristics such as functional disability (ADL and IADL scores) or the number of comorbidities (Charlson comorbidity Index). The reason for this lack of correlations remains unknown, and the relationship between PSS and these clinical characteristics needs to be further studied.

Additionally, it should be noted that in our study, we examined the correlation between perceived stress and other clinically relevant outcomes such as post-dialysis fatigue (PDF), both in terms of prevalence (PDF yes:no) and characteristics (PDF intensity, PDF duration, PDF frequency). Furthermore, we evaluated whether there is a correlation between PSS and the time of recovery after dialysis (TIRD). We found that there is no correlation between PDF prevalence/characteristics and PSS, nor between TIRD and PSS. This is a new and partly unexpected finding because it is known that stress in everyday life can be a cause of tiredness. Nevertheless, it is well known that the pathogenesis of PDF and long TIRD remain poorly understood due to small, outdated studies, lack of control groups for comparison, and largely observational data. Overall, the data point to hemodynamic and osmotic effects of the dialysis treatment itself, as well as psychological factors contributing to fatigue, but causes have not been definitively identified [37].

Finally, it may be considered that confounding factors, such as individual differences in social support or pre-existing psychological conditions, could be considered as potential mediators and deserve more attention.

In the current study, we did not measure quality of life, but in other chronic diseases, high perceived stress is associated with worse quality of life. In another study on HD patients, health-related quality of life was found to explain the variability of the PSS score [3]. Future studies should investigate the relationship between perceived stress and quality of life in HD patients, considering both general and health-related quality of life dimensions.

The current findings raise a question that should be taken into consideration by research and services that deal with psychological interventions dedicated to patients with chronic disease: is there a role for the treatment of perceived stress in these patients? For instance, a study showed that mindfulness meditation can significantly decrease perceived stress [38]. Garcia-Martinez and colleagues [3] showed that in HD patients, PSS levels negatively correlate with the development of resilience, defined as a coping style that allows for positive progress in adverse situations [39]. Thus, the development of interventions to promote resilience also could be implemented to reduce perceived stress and increase the well-being of these patients. In addition to mindfulness meditation and interventions to promote resilience, other non-pharmacological treatments have been proposed to ameliorate symptoms of depression in HD patients, such as cognitive-behavioral therapy (CBT), physical exercise, music therapy, light therapy, and healthy diet [35]. Among the most effective practical interventions, psychological support through CBT has been shown to improve depressive symptoms, anxiety, and quality of life in HD patients [40]. Also, promoting a healthy lifestyle, including moderate physical activity tailored to the patient’s condition, has proven beneficial for both physical health and symptoms of depression [41].

Considering the study limitations, the current research is not without caveats: firstly, the sample includes only Italian patients derived from one center in Italy; secondly, being observational and crossover, no conclusion can be drawn about causality; thirdly, this study did not evaluate the relationship between PSS and quality of life, despite this connection being reported in other chronic diseases. Future studies should therefore incorporate this analysis into their research.

## 5. Conclusions

This study highlights the significant prevalence of moderate to high perceived stress among HD patients, particularly in women, and its strong association with depression. These findings emphasize the critical need for targeted interventions addressing stress management, especially gender-specific approaches. Future research should explore the connection between perceived stress and quality of life to develop comprehensive strategies for improving psychological outcomes in this vulnerable population. The absence of a correlation between perceived stress and clinical outcomes, such as post-dialysis fatigue, time to recovery, and functional markers suggest the need for future research to more thoroughly explore the interactions between psychological and clinical variables.

## Figures and Tables

**Table 1:** Demographic, clinical, and laboratory characteristics of patients included in the study. Data are expressed as median [95% Confidence Intervals, CI]. HD, bicarbonate hemodialysis; HDF, hemodiafiltration; ADL, activities of daily living; IADL, instrumental activities of daily living; PDF, Post-dialysis fatigue; TIRD, Time to recovery after dialysis.

Variables	All Patients	Male Patients	Female Patients	*p*
Total number of patients	223	144	79	
Age (years)	70 [68–72]	69 [66–71]	63 [68–76]	0.067
Sex (M:F)	144:79			
▪ Primary cause of ESRD Hypertension Diabetes Glomerulonephritis Polycystic renal disease Interstitial nephritis Unknown	64 (26%) 56 (26%) 49 (22.2%) 28 (13%) 15 (7.4%) 11 (5.5%)	39 37 31 19 11 7	25 19 18 9 4 4	0.954
Dialytic age (months)	57 [48–70]	56 [48–70]	60 [46.4–63.8]	0.863
HD: HDF	153:70	101:48	52:22	0.760
ADL	6 [6–6]	6 [6–6]	6 [6–6]	0.172
IADL	8 [8–8]	8 [8–8]	8 [7–8]	0.231
Charlson comorbidity index	4 [4–5]	4 [4–5]	4 [3–5]	0.353
BDI	10 [9–11]	8.5 [7–10]	12 [10–15]	0.0001
IDWG (kg)	2.2 [2–2.5]	2.5 [2.2–2.8]	2 [1.6–2]	0.0001
PDF (NO:YES)	104:132	73:71	28:51	0.034
PDF intensity	2 [1–3]	1 [0–2]	3 [2–3]	0.007
PDF duration	2 [1–2]	1 [0–2]	3 [1.7–3]	0.049
PDF frequency	1 [0–2.5]	1 [0–2]	3 [1–3]	0.109
PDF summary	7 [3–8]	4 [0–8]	9 [6–9]	0.039
TIRD	120 [30–120]	45 [0–120]	180 [1220–240]	0.011
Serum albumin (g/dL)	3.4 [3.3–3.5]	3.4 [3.3–3.5]	3.4 [3.3–3.5]	0.408
Serum creatinine (g/dL)	9.1 [8.6–9.6]	9.7 [9.1–10.3]	8.3 [7.7–8.8]	0.0001
Hemoglobin (g/dL)	11 [10.9–11.3]	11.1 [10.8–11.4]	11 [10.6–11.3]	0.731
Hematocrit%	34 [33.5–34.8]	34.1 [33.3–35]	34 [33–35.1]	0.832

**Table 2:** Perceived stress and demographic, clinical, and laboratory variables. Data are expressed as median [95% Confidence Intervals, CI].

	Perceived Stress Scale			
	LOW (0–13) (n. 40)	MODERATE (14–26) (n. 158)	HIGH (27–40) (n. 25)	*p*-Value
Age (years)	67 [64.1–75]	70 [68.5–69]	77 [66–80]	0.094
Sex (M:F)	31:9	101:57	12:13	**0.014**
Dialytic age (months)	57.5 [54–64]	56 [47–58]	70 [36–73.5]	0.857
HD: HDF	27:12	107:51	18:7	0.906
ADL	6 [1–6]	6 [0–6]	6 [0–6]	0.224
IADL	8 [1–8]	8 [0–8]	7 [0–8]	0.190
Charlson comorbidity index	4 [3–4]	4 [5–5]	4 [5–5]	0.358
BDI	9 [8–9]	10 [8–10]	20 [12.5–24]	**0.0003**
IDWG (kg)	2 [1.6–2.7]	2.4 [2.1–2.7]	2 [2–2.6]	0.271
PDF (NO:YES)	18:22	74:84	11:14	0.985
PDF intensity	1 [0–3]	2 [0–2]	2 [0–4]	0.814
PDF duration	1 [0–3]	2 [0–2]	2 [0–4]	0.888
PDF frequency	1 [0–4]	2 [0–3]	1 [0–3]	0.656
PDF summary	3 [0–10]	8 [0–8]	7 [1.5–10]	0.782
TIRD	15 [0–180]	120 [0–60]	120 [0–600]	0.442
Serum albumin (g/dL)	3.6 [3.5–3.6]	3.4 [3.3–3.4]	3.6 [3.1–3.4]	0.120
Serum creatinine (g/dL)	9.6 [8.9–11]	9.4 [8.7–10.3]	8.4 [6.5–8.9]	0.094
Hemoglobin	11.3 [10.8–11.9]	11 [10.9–11.5]	11 [9.9–11.3]	0.210
Hematocrit%	34.1 [33.6–36.2]	34 [33.2–35]	34 [31.4–36]	0.397

**Table 3:** Multivariate regression analysis results revealed a significant association between the three groups of PSS scores and the Beck Depression Inventory (BDI) score.

						95% CI	
	Coefficient	SE	t	*p*	r	Lower Bound	Upper Bound
BDI	0.016	0.004	3.70	<0.001	0.243	0.007	0.025
Age	0.0007	0.0027	0.255	0.798	0.017	−0.005	0.006
Sex	0.100	0.076	1.323	0.187	0.089	−0.049	0.251
Serum creatinine	−0.010	0.013	−0.758	0.449	−0.051	−0.038	0.017

**Table 4:** Distribution of patients according to the PSS score and the relative BDI score for each group. Upper panel: all patients (*p* = 0.004); Middle panel: male patients (*p* = 0.005); lower panel: female patients (*p* = 0.098).

	Perceived Stress Scale (PSS) Score		
	Low (n. 40)	Moderate (n. 158)	High (n. 25)
BDI < 17	31 (77.5%)	111 (70.3%)	10 (40%)
BDI ≥ 17	9 (22.5%)	47 (29.7%)	15 (60%)
	Low (n. 31)	Moderate (n. 101)	High (n. 12)
BDI < 17	28 (90.3%)	85 (84.1%)	6 (50%)
BDI ≥ 17	3 (9.7%)	16 (15.9%)	6 (50%)
	Low (n.9)	Moderate (n. 57)	High (n. 13)
BDI < 17	7 (77.7%)	43 (75.4%)	6 (46.2%)
BDI ≥ 17	2 (22.3%)	14 (24.5%)	7 (53.8%)

## Data Availability

The datasets generated during and/or analyzed during the current study are available from the corresponding author on reasonable request.
